# Identification of the Differential Effect of City-Level on the Gini Coefficient of Health Service Delivery in Online Health Community

**DOI:** 10.3390/ijerph16132314

**Published:** 2019-06-29

**Authors:** Hai-Yan Yu, Jing-Jing Chen, Jying-Nan Wang, Ya-Ling Chiu, Hang Qiu, Li-Ya Wang

**Affiliations:** 1 School of Economics and Management, Chongqing University of Posts and Telecommunications, Chongqing 400065, China; 2 Department of Statistics, Eberly College of Science, The Pennsylvania State University, University Park, PA 16802, USA; 3 College of International Finance and Trade, Zhejiang Yuexiu University of Foreign Languages, Shaoxing 312000, China; 4 College of International Business, Zhejiang Yuexiu University of Foreign Languages, Shaoxing 312000, China; 5 School of Computer Science and Engineering, University of Electronic Science and Technology of China, Chengdu 611731, China; 6 Big Data Research Center, University of Electronic Science and Technology of China, Chengdu 611731, China

**Keywords:** inequality, health service, causality, medical specialty, consultation

## Abstract

Inequality of health services for different specialty categories not only occurs in different areas in the world, but also happens in the online service platform. In the online health community (OHC), health services often display inequality for different specialty categories, including both online views and medical consultations for offline registered services. Moreover, how the city-level factors impact the inequality of health services in OHC is still unknown. We designed a causal inference study with data on distributions of serviced patients and online views in over 100 distinct specialty categories on one of the largest OHCs in China. To derive the causal effect of the city-levels (two levels inducing 1 and 0) on the Gini coefficient, we matched the focus cases in cities with rich healthcare resources with the potential control cities. For each of the specialty categories, we first estimated the average treatment effect of the specialty category’s Gini coefficient (SCGini) with the balanced covariates. For the Gini coefficient of online views, the average treatment effect of level-1 cities is 0.573, which is 0.016 higher than that of the matched group. Similarly, for the Gini coefficient of serviced patients, the average treatment effect of level-1 cities is 0.470, which is 0.029 higher than that of the matched group. The results support the argument that the total Gini coefficient of the doctors in OHCs shows that the inequality in health services is still very serious. This study contributes to the development of a theoretically grounded understanding of the causal effect of city-level factors on the inequality of health services in an online to offline health service setting. In the future, heterogeneous results should be considered for distinct groups of doctors who provide different combinations of online contributions and online attendance.

## 1. Introduction

**Figure 1:**
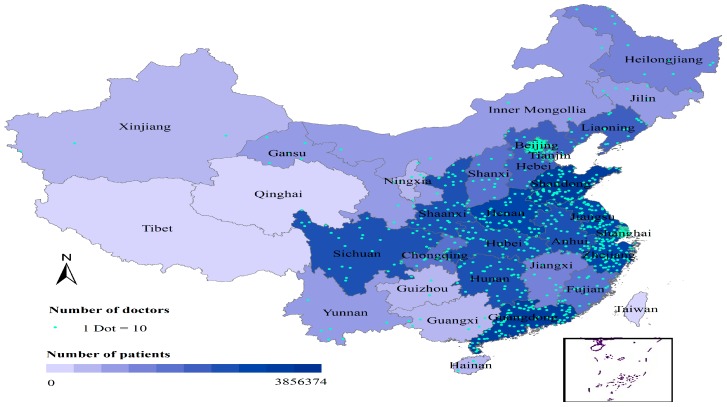
Unequal geograpghical distribution of medical resources in the investigated online health community. Beijing and Shanghai are the cites (city level = 1) with richer healthcare resources (including a larger population of doctors) and patients than those of the other cities (city level = 0). The size of circles indicates the number of patients, and the darkness of the color in the circles indicates the number of doctors. Data were collected from the online health service platform www.haodf.com on 26 June 2017.

With the development of health services worldwide [1], the inequality of health services for different specialty categories not only occurs in different areas, but also happens in online services, i.e., rural-urban health disparities [2]. More importantly, substantial inequalities remain in the geographical distribution of medical resources (as illustrated in Figure 1). In particular, provinces in western China have the lowest levels of health resources [3]. With its potential to mitigate the low levels of medical resources in rural areas, the online health community (OHC) concept has become a physician-patient communication platform [4] and a site for the public to share physician reviews. Up to 500,000 people with chronic diseases have used PatientsLikeMe [5], an online health service in USA, according to a report by The Economist [6]. However, few studies have focused on the inequality of online health services, especially in the inequality of health services for different city-levels. As our previous studies suggested [4], physicians with more past physician online contributions, with higher review ratings, and not coming from cities with rich healthcare resources, were more willing to participate in OHC activities. The city-level (or state level) has been studied in other areas, i.e., equity in health [3] and public health [7]. However, the causal effect of the city-level on the inequality of health service is still unknown, especially for online healthcare communities.

As our previous findings [4] suggested, in various specialty areas, the average levels of physician online contribution are different. Even after the characteristics associated with the potential outcomes are controlled for differences in observed characteristics, there are reasons to believe that the treated and untreated differ in unobservable characteristics [8,9]. In this scenario, the treated and untreated may not be directly comparable, even after adjusting for observed characteristics. The city-level is an important factor that aggregates the information of geographical distribution and other related resources distribution [10,11]. Can we still identify and estimate the causal effects of the (city-level) characteristics on the inequality of health service between online views and offline serviced patients for specialty categories? To find a solution to those issues, we designed a causal inference study to examine the average treatment effect (ATE) of the city-level, identifying the difference of inequality of health service between online views and offline serviced patients for specialty categories.

### 1.1. Research Issues

Although the provision of OHCs can mitigate the low levels of medical resources in rural areas, few studies have focused on the inequality of online health services, especially on the inequality of health services for different specialty categories. The OHC platform can be regarded as an online-to-offline (O2O) system that provides both communication channels (interaction) for online medical services and records (or feedback) for offline medical services. Although many pieces of research have suggested a long tail phenomenon exists in the online product sale platform, seldom have they simultaneously taken both the inequalities in online views and in the offline service (patients’ consultation) into consideration. This study attempts to bridge this knowledge gap. We examine whether the online health community reduces the inequality of health service for different specialty categories through a retrospective study of the Lorenz curve of doctors’ service diversity. Our motivation is trying to answer the following issues: (1) What kind of patterns characterize the distribution of medical service delivery in distinct specialty categories in the online health community? (2) How does the factor ‘city-level’ impact the inequality of health services in OHCs? (3) How to identify the difference of the response of the Gini coefficient with the treatment variable of the city-level and other confounding variables?

### 1.2. Literature Review

Among OHC platform users, the three types of services with the highest usage rate [4] are medical information inquiry (10.8%), online registration (10.4%), and online consultation services (6.4%) [12]. Meanwhile, the online health community can also have the facilities, including guiding the patients to go to hospitals for necessary conditions and multiple virtual visits with their doctors to save time, travel costs and avoid environmental pollution [13]. As the posters of Good Doctor (the OHC with the largest population of registered doctors in China) online platform says “based on patients’’ self-introduction of their conditions, those comments presented by doctors can only be deemed as references rather than direct guidelines for diagnosis and treatment”. Since patients often seek information (doctor’s outpatient time, their personal introduction and review rating, etc.) about doctors on the OHC, they also revisit the community to give feedback (i.e., ratings, online registration, thank-you letters, and gifts) to their doctors after the face to face medical service. Although many studies have suggested a long tail phenomenon exists in online product sale platforms [14,15] and online and offline prices are similar [16], few of them took the inequalities of doctors’ service delivery (online or offline service) into consideration.

Studies have investigated the influence of the general structure of inequality on the service value and practices in society. One study [15] investigated the inequality of online sales in recommender systems. Focusing on the distribution of its demand and revenue, their study associated the average influence of the network on each category with the inequality, and quantified the inequality using the Gini coefficient. Using ordinary least-squares regression, the study [17] estimated the association between a category’s Gini coefficient (RevenueGini) and the average PageRank of its books (AvgPageRank). This paper is among the first to measure the concentration of health service delivery in OHCs.

The Lorenz curve was first proposed as a graphical statistic tool to express the concentration of wealth in a population in 1905 [18]. Thus one can select any quantile to characterize concentration. Alternatively, the Gini coefficient [1] as a summary index of concentration was frequently applied for studying the concentration of income in a population and had been implemented to many problems. Recently, the Lorenz curve and Gini coefficient have also seen applications in the area of health and medical services research. For example, the Lorenz curve and Gini coefficient have been implemented to explore the relation between distribution of health professionals and the distribution of patients [18]. On the basis of some quantity of interest, the inference of both the Lorenz curve and the Gini coefficient involves ranking the units of observation and estimating cumulative proportions.

A number of approaches are capable of revealing the associative relationships between the outcomes and the related independent variables at a significant statistic level. The causal inference method takes the advantages of non-significant related covariates, which assigns treatment experiments on different units. However, challenges lie in the identification of the population average causal effect of the treatment on the dependent variables. Average treatment effect is a term which refers to the measure comparing treatments (or policies) in randomized experiments [19]. Although treatment effect originated in the medical literature concerned with the causal effects of binary treatments, such as drug trials, the term is now applied more generally, such as evaluation of policy interventions and dynamic treatment regimes. In a randomized experiment, the ATE can be estimated using a differential comparison in mean outcomes for treated and untreated units. However, the ATE is generally deemed as a causal parameter of interest. With random assignment mechanism, both observational studies and experimental study designs may enable the investigator to estimate an ATE in a variety of methods. When the ATE is estimated by the difference between these two averages, it is also an estimate of the central tendency of the distribution of unobservable individual-level causal effects [20]. With a sample randomly constituted from a population, the ATE from the sample (the SATE) can asymptotically converge to the population ATE (or PATE) [21]. The primary goal of causal analysis is to explore the selected effects of a particular cause, rather than the search for all possible causes of a particular outcome or other relative effects. The rise of the counterfactual inference model has increased the popularity of data analysis procedures which are most clearly useful for the discovery of causality. For a saturated regression model, the lack of covariate balance will be revealed to the investigator when the regression routine drops the coefficient for the zero cells. However, if a constrained regression model were fit, such as if covariates were modelled as a simple linear term interacted with treatment, the regression model would yield seemingly reasonable coefficients. Under unconfoundedness or exogenous treatment assignment, estimation of ATE is often hampered by a lack of covariate balance. This lack of covariate balance leads to imprecise estimates and often makes estimators sensitive to the specification of models. With observational data, it is desirable for influencing causal effects to replicate a randomized experiment by obtaining covariate balance between the treatment and control groups. This goal of randomization can often be obtained through choosing well-matched samples of the original treatment and control units, thereby reducing bias due to the confounding covariates [22]. In such a context, researchers have often used informal methods for trimming the sample [23]. To find the region of overlap, the propensity score method may not capture all dimensions of the common support; subsequent matching is implemented to achieve covariate balance [20].

## 2. Materials and Methods

### 2.1. Research Models

In the research design, the treatment variable (city-level) represents the doctor’s location status at a specific time. Second, the mean and variance of the number of doctors’ articles across the specialty categories, mean in the degree of voted diversity, mean of doctors’ review rating and mean in doctors’ online contribution as independent variables are considered as the covariates. Based on this framework, we can verify whether doctors’ average treatment effect of cities with rich healthcare resources on the inequality of health service is the same for online service (online reviews) and offline service delivery (serviced patients) in different specialty categories.

Gini coefficient [24] was introduced to reveal the distributions (patterns) within categories in a way that is comparable across doctors’ specialty areas by calculating the Gini coefficient of each category of the doctors’ online service. In applications, the Gini coefficient frequently accompanies a graphical presentation of the Lorenz curve. To comparative analyses of the inequalities in service delivery of online service and in the offline service delivery, we defined two concepts with the Gini coefficient, Gini coefficient of service delivery and Gini coefficient of patient reviews.

The difference of Gini coefficients (of serviced patients or online views) was the dependent variable of interest, and the average number of articles, average breadth of service diversity, average doctor review rating and average doctor online contribution are set as the covariate variables and the city-level ($T_{i}$) as the treatment variable. The treatment variable is a binary (0–1) variable, which represents the doctors staying the cities of rich healthcare resources or not at the data acquisition time. The treatment variable is employed to test the average treatment effects of their status. For example, for all the specialty categories, the statistical analysis is designed and conducted for those doctors from cities of rich healthcare resources (i.e., Beijing and Shanghai) $T_{i}=1$ and (other cities in China) $T_{i}=0$, respectively. The choice of Beijing and Shanghai is based on two aspects. First, the healthcare resources in those two cities are much richer than those in other cities or even provinces in China. Approximately 22% of all physicians are working in Beijing or Shanghai, the two largest cities in China. This naturally reflects the relative inequality of the health service of medical resources in large cities. In all the 31 regions, Shanghai ranked first on the perspective of health care institutions (number per 10,000 km^2^), health technical personnel, beds in health care institutions and health investment, while Beijing occupied the second place [25]. Second, those two cities are often formally treated as special cases, compared to any other cities in China. One study [26] revealed that Shanghai with the highest level of economic development had more advanced computed tomography and magnetic resonance imaging machines, and higher government subsidies on these two types of equipment.

The average treatment effects study has many strengths. First, this model will avoid selection bias in the estimation of treatment effects. The bias problem is critical for analyzing imbalanced data, i.e., the distribution of numbers of owning $T_{i}=1$ is not overlapped with that of owning $T_{i}=0.$ Second, although other independent variables may attract the readers on the topic of this area, the average treatment effects of city-level ($T_{i}$) in the inequality of health service attract the most important concerns in the OHC stakeholders. The definitions and measurements of all variables are listed in Table 1.

With the two dependent variables, we can estimate the doctors’ average treatment effect of cities of rich healthcare resources on the inequality of health service in different specialty categories separately and compared them between online views and offline service (patients).

### 2.2. Materials

Through web crawler technology, data from the Good Doctor website were collected (on 26 July 2017) and filtered for the purposes of the study. In the data set of 142,448 samples, 140,344 doctors with personal homepages were commonly considered to be genuinely involved in this OHC. The collected data set contained all the values of this study as well as the doctor’s identity document (personal web site) and other de-identified information. The following filtering criterion was set to design an observational retrospective study; (a) Amount of served patients for doctor $i^{\prime }$ is larger than 0, and the volume of patient online reviews for doctor $i^{\prime }$ is larger than 0. (b) The number of doctors’ articles is larger than 0, number of reviews rating larger than 0, doctor $i^{\prime }$ is online contributions larger than 0 and the number of patients’ votes larger than 0.

**Figure 2:**
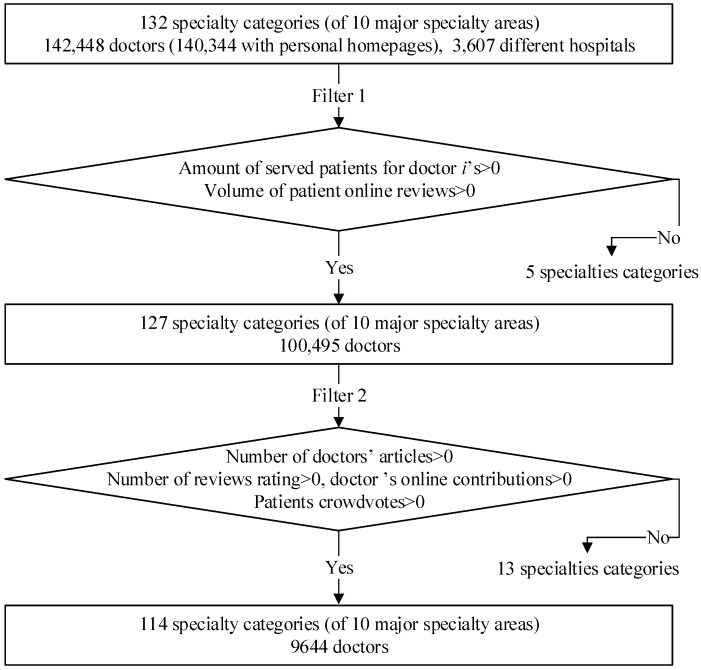
Data Acquired and Filtering Process (Accessed From Good Doctor Website).

After filtering, 9644 samples of doctors remained from the original data set. Meanwhile, 114 specialty categories were filtered from the original 132 categories. The data acquired and filtering process is illustrated in Figure 2.

The filtered samples have the following characteristics. First, our samples were from a large heterogeneous population with diverse backgrounds. The 9644 doctors came from 127 different specialty categories and 1338 different hospitals widely distributed throughout China. Second, the number of service deliveries and the number of patient reviews were collected for the retrieved doctors on the OHC. Although their usage time was different, the corresponding values of the independent variables were also collected during the same period for their usage time. Third, the number of doctors’ articles were collected without distinguishing between the original articles and reprinted long articles (not the communication posts with patients). We also collected the doctors’ review ratings (regarded as online word-of-mouth) from the stars labeled on the OHC. The average score of these ratings is 2.756 for all the sample data on a scale from 1 (the lowest) to 5 (the highest). Moreover, despite the association with the posted articles on the website, the contribution scores of the doctors were also impacted by many other factors, including the posted articles communicating with the patients online on the website. The other values we collected were the patient votes, which were different from the doctors’ review votes for the word-of-mouth rating and the case records of doctors’ accumulated clinical experience. Finally, the values of the location of hospitals were also collected for those doctors clustered in the samples. After filtering, 2603 (27%) of all the doctors were from Beijing or Shanghai, which are China’s two largest developed cities (level-1), while the other 7041(73%) doctors from the other cities (level-0). Thus, a causal inference study can be designed with those collected and filtered data samples.

### 2.3. Measures

Before examining the OHC platform’ effects, it is necessary to distinguish between service delivery and service diversity. Service diversity typically measures how many different services a doctor offers. It is a supply-side measure of breadth. In contrast, we use the diversity of service delivery to describe the concentration of market shares conditional on doctors’ assortment decisions [27].Here we introduce the Gini Coefficient to quantify the concentration of service delivery in OHC, because the Gini index is much easier for us to understand the distribution of inequality as a ratio of two areas in Lorenz curve diagrams than the other inequality metrics (i.e., S-Gini index) [24].

#### 2.3.1. Gini Coefficient: Quantifying the Distribution of Service Inequality

To identify the causal effect of cities of rich healthcare resources on service inequality, our research framework is designed as a retrospective observational study. We aim to investigate the outcomes from two aspects: (a) Gini coefficient of service delivery: offline registered patients, and (b) Gini coefficient of patient reviews: online service. Thus, the dependent variable will be used to reveal the patterns (i.e., inequality phenomena) of the doctors’ online service and reveal the relationship between specialty category’s Gini coefficient (SCGini) and doctors’ endorsement on a diversity of specialty categories.

Let L(p) be the Lorenz curve [24] denoting the percentage of the provider’s service delivery generated by the lowest (100×p)% of doctors clustered in the same specialty area during a fixed time period. In our analysis, the Lorenz Curve L(p) is drawn inside a square box with the x-axis being a cumulative percentage of doctors’ serviced patients (service delivery) and the y-axis being the cumulative percentage of service delivery for doctors clustered in the same specialty area during a fixed time period. The Lorenz curve of a category’s service delivery ranks the services (online medical consultation) in increasing order of the amount of past served patients, then plots the cumulative fraction L(p) of the amount of service delivery (served patients) associated with each ascending rank percentile *p*, where 0 < *p* < 1.

This study on the total amount of doctor i’s past served patients online will provide evidence to factors of success on which the potential customers select an online doctor and reveal the evolving mechanism of clinical acceptance of telemedicine. $SP_{i}$. is measured as the cumulative size of the served patients (referring to the doctors’ service delivery) in the past. Therefore, the volume of service delivery for doctors clustered in the same specialty area during a fixed time period, $SP_{j}$ is calculated by summing the total amount of past served patients of all the doctors in the same specialty area:

(1) $$SP_{j}=\overset{N_{j}}{\underset{i=1}{\sum }}SP_{i}\left(j\right)$$

where $SP_{i}\left(j\right)$ is the total amount of doctor i’s past served patients online in the specialty category (discipline) *j*, $N_{j}$ is the number of doctors clustered in the specialty category j.

Thus, the Gini coefficient of distribution of service delivery SCGini defined by [15]. The Gini coefficient SCGini measures the distributional inequality of the amount of service delivery (serviced patients). The SCGini of serviced patients for the specialty category j is defined as:

(2) $$SCGini_{j}\left(SP\right)=\frac{Area\left(SC_{j},45^{{}^{\circ}}\right)}{0.5}$$

(3) $$Area\left(SC_{j},45^{{}^{\circ}}\right)=\int_{0}^{1}\left(p-L\left(p\right)\right)dp$$

where $Area\left(SP_{j},45^{{}^{\circ}}\right)$ is the area between the Lorenz Curve of service delivery and a $45^{{}^{\circ}}$ line. Thus, SCGini measures how much L(p) deviates from the $45^{{}^{\circ}}$ line, SCGini∈[0,1]. A value SCGini = 0 reflects diversity (all services have equal service delivery), whereas values near one represent concentration (a small number of services account for most of the service delivery).

When service delivery is perfectly evenly distributed among products, the Lorenz Curve L(p) coincides with a $45^{{}^{\circ}}$ line and the Gini Coefficient SCGini equals zero. As the distribution becomes more concentrated, the L(p) curves away from a $45^{{}^{\circ}}$ line and the SCGini increases. Thus, SCGini is an aggregate inequality measure and vary anywhere from 0 (perfect equality) to 1 (perfect inequality). Perfect equality in our case illustrates that all the doctors in that category (specialty area) have the same number of service delivery, and perfect inequality illustrates one doctor in the category service all the patients in that specialty area and all other doctors in the category have zero of served patients.

Similar to the definition of $SCGini_{j}\left(SP\right)$, the Gini coefficient SCGini measures the distributional inequality of the number of patient reviews for the doctors in the sociality category.

First, the volume of patient online reviews for doctors clustered in the same specialty area during a fixed time period, $OR_{j}$ is calculated by summing the total amount of past online reviews $OR_{i}$(*j*) of all the doctors in the same specialty area:

(4) $$OR_{j\;}=\overset{N_{j}}{\underset{i=1}{\sum }}PR_{i}\left(j\right)$$

where $PR_{i}\left(j\right)$ is the total amount of doctor i’s past patients reviews for doctor *i* in the specialty category (discipline) *j*, $N_{j}$ is the number of doctors clustered in the specialty category *j*.

SCGini of patient reviews for the specialty category *j* is defined as:

(5) $$SCGini_{j}\left(OR\right)=\frac{Area\left(OR_{j},45^{{}^{\circ}}\right)}{0.5}$$

A value SCGini (OR) = 0 reflects diversity (all doctors have equal online reviews), whereas values near one represent concentration (a small number of doctors account for most of the online reviews).

#### 2.3.2. Measures of Doctors’ Endorsement

To test this main conjecture, we use the mean and variance of the number of doctors’ articles across the specialty categories, mean in the degree of voted diversity, mean of doctors’ review rating and mean in doctors’ online contribution as independent variables.

a) Mean of the number of doctors’ articles:

In this study, we measured the number of doctors’ articles through a cumulative count of the articles of each doctor listed on the Good Doctor website. $NDAMea_{j}$ is measured as the mean of the number of doctors’ articles for doctors clustered in the specialty category j:

(6) $$NDAMea_{j}\;=\;\frac{\sum_{i=1}^{N_{j}}NDA_{i}\left(j\right)}{N_{j}}$$

where $NDA_{i}\left(j\right)$ is the number of doctors’ articles of the doctor i clustered in the specialty category j, $N_{j}$ is the number of doctors clustered in the specialty category j.

b) Degree of voted diversity:

Given the voting states $\left(S_{i},\;Votes\left(S_{i}\right)\right)$, $S_{i}=\left\{S_{i1},S_{i2},\ldots ,S_{im}\right\}$ is the vector of doctor i’s service specialty labeled by the serviced patients in specialty category j, and $Votes\left(S_{i}\right)$ is the corresponding volume vector of their votes. The total amount of doctor i’s service specialties labeled by the serviced patients:

(7) $$BVS_{i}\left(j\right)\;=\;\overset{m}{\underset{j=1}{\sum }}1_{\left(\;Votes\left(S_{i}\right)>0\right)}$$

$BVSMea_{j}$ is measured as the average breadth of the voted specialties (from patient votes) of all the doctors clustered in specialty category j.

(8) $$BVSMea_{j}\;=\;\frac{\sum_{i=1}^{N_{j}}BVS_{i}\left(j\right)}{N_{j}}$$

where $BVS_{i}\left(j\right)$s the breadth of the voted specialties (from patient votes) of the doctor *i* in specialty category *j*, and $N_{j}$ the number of doctors clustered in the specialty category j.

c) Mean of the doctors’ review rating:

In this study, we measured the physicians’ ratings in user reviews through the star scores listed on the Good Doctor website. $DRRMea_{j}$ is measured as the mean of the ratings in user reviews of the doctors clustered in the specialty category j:

(9) $$DRRMea_{j}=\frac{\sum_{i=1}^{N_{j}}DRR_{i}\left(j\right)}{N_{j}}$$

where $DRR_{i}\left(j\right)$ is the ratings in user reviews of the doctor i clustered in the specialty category $N_{j}$ is the number of doctors clustered in the specialty category j.

d) Mean of the doctors’ online contribution:

Essentially, the existence of online contributions means that members are involved in community-related activities, such as sharing information actively, responding positively to other members’ questions, and intuitively interacting with other members [16,19]. In this study, we measured the physicians’ online contribution through the contribution scores listed on the Good Doctor website. There are three principal ways in which the contribution score can change. First, when physicians update their personal information, such as outpatient information and consultation range, in a timely manner, their contribution scores can be increased through the OHC administrator’s audit. Second, physicians are encouraged to post medical articles for patients on the website. After the article is referenced by the Good Doctor website, the contribution score is updated. Third, if a physician can answer a patient’s question online, his/her contribution score will be increased. $DOCMea_{j}$ is measured as the mean of the contribution score for the doctors clustered in the specialty category j:

(10) $$DOCMea_{j}=\frac{\sum_{i=1}^{N_{j}}DOC_{i}\left(j\right)}{N_{j}}$$

where $DOC_{i}\left(j\right)$ is the contribution score for the doctor i clustered in the specialty category $N_{j}$ is the number of doctors clustered in the specialty category j.

#### 2.3.3. Propensity Score: Measure of the Likelihood Being Treated

The propensity score is often employed to reduce the dimensionality of the causal influence problem. The propensity score is the conditional probability of assignment to a particular treatment gen a vector of observed covariates [28].

Let $p\left(X_{i}\right)$ be the probability of unit i having been assigned to treatment, and the propensity score was defined as [29]:

(11) $$p\left(X_{i}\right)=\mathrm{Pr}\left(T_{i}|X_{i}\right)=E\left(\left(T_{i}|X_{i}\right)\right)$$

where $\mathrm{Pr}\left(T_{i}|X_{i}\right)$ is the probability of being assigned to the treatment given $X_{i}$ and E(x) is the expectation operator of *x*, $T_{i}$ is a dummy variable with two levels as detailed in Table 1. Here $X_{i}$ denotes the covariates, i.e., $NDA_{i}$, $DRR_{i}$ and $DOC_{i}$. Usually, the propensity score was estimated by training the logistic regression [29]:

(12) $$logit\left(T_{i}\right)=\beta_{0}+\beta_{1}NDA_{i}+\beta_{2}BVS_{i}+\beta_{3}DRR_{i}+\beta_{4}DOC_{i}+\varepsilon_{t}$$

where $\beta_{0}$ is the coefficient of the constant term and $\beta_{j}$ = 1, 2, 3, 4, are the coefficients of control variables as detailed in Table 1. The error term $\varepsilon_{i}$ obeys normal distribution with mean 0 and variance $\sigma^{2}$.

To achieve a balanced control-treatment case dataset, matching on pre-treatment covariates is one popular method. We match control-treatment cases on pre-treatment covariates with the propensity score. In the matching process, the scalar can be preset for the number of matches which should be found, i.e., the default value 1 is for one-to-one matching. More similar units are more likely to experience more similar trends so the parallel path assumption may be more plausible. Finally, we run the causal effect regression model with the matched data-set.

### 2.4. Statistical Analyses

Having defined our two main variables—service diversity and Gini—we now turn to our empirical analysis. To test the main conjecture of whether doctors’ patient votes will affect service usage, it’s easy to think about the associative relationship between the covariates and the outcomes. We first fit these data for ten specialty areas by examining how an increase in its influence might enhance or diminish the long tail of medical service demand, rather than fit the size of serviced patients and scale of vote data for the individual doctors. However, we are not only investigating the associative relationship of main effects but also revealing the causal effect of the treatment variable on the outcome, the inequality of health service for different specialty categories.

To further reveal the causal effect, the statistical analysis is designed and conducted for those doctors, respectively. The average treatment effect [19] refers to the causal effect of the binary (0–1) variable T on an outcome variable of interest, comparing their average outcomes with sample covariate balanced:

(13) $$ATE\left(SCGini_{j},T\right)=E\left(SCGini_{j}\left(T=1\right)-SCGini_{j}\left(T=0\right)\right)$$

where E(x) is the expectation of *x*.

For all the specialty categories, the $SCGini_{j}$ consists of two aspects, the specialty category’s Gini coefficient of serviced patients and the specialty category’s Gini coefficient of online reviews. Those results will be employed to verify the effectiveness of online service and offline service.

In the form of regression [20], the causal effect α can be a model with the linear model:

(14) $$Y_{j}=\mu +\alpha T_{j}+\beta X_{j}+\varepsilon_{j}$$

where $Y_{j}$ denotes the outcomes of the jth units, namely, the Gini coefficient of the j-th categories; $T_{j}$ the indicator of treatment variable, and $X_{j}$ the covariates and $\varepsilon_{j}$ the error for unit j.

The coefficient for the treatment indicator α still represents the average treatment effect, but controlling for covariates can improve the efficiency of the estimate. More generally, the regression can control for multiple covariate predictors. As the covariates can be substituted by the observational variables, the causal inference using regression [20] on the treatment variable can be formed as:

(15) $$Ln\left(SCGini_{j}\right)\;=\;\mu \;+\;\alpha T_{j}\;+\;\left[\begin{array}{c} \beta_{1}Ln\left(NDAMea_{j}\;\right)\;+\;\beta_{2}Ln\left(BVSMea_{j}\right)+\\ \beta_{3}Ln\left(DRRMea_{j}\right)\;+\;\beta_{4}Ln\left(DOCMea_{j}\right) \end{array}\right]\;+\;\varepsilon_{j}$$

where $Y_{j}$ is substituted by $Ln\left(SCGini_{j}\right)$, the logarithm transform of the Gini coefficient of patients or views.

## 3. Results

### 3.1. Overlap of the Confounding Variables

With the propensity score matching theory [30], we analyzed the experimental data using logistic regression (10) with one main effect (on treatment) for each covariate. The nearest neighbor method was implemented to achieve control cases to the focus cases.

First, as the literature usually has done [22,31], graphical diagnostics are helpful for quickly assessing the covariate balance. The histogram distributions of propensity scores in the original and matched groups are also useful for assessing common support. Although the densities of raw treatment and matched treatment cases did not change, those of raw control and match controls took significantly changes. The results show an adequate overlap of the propensity scores, with a good control match for each treatment unit.

**Figure 3:**
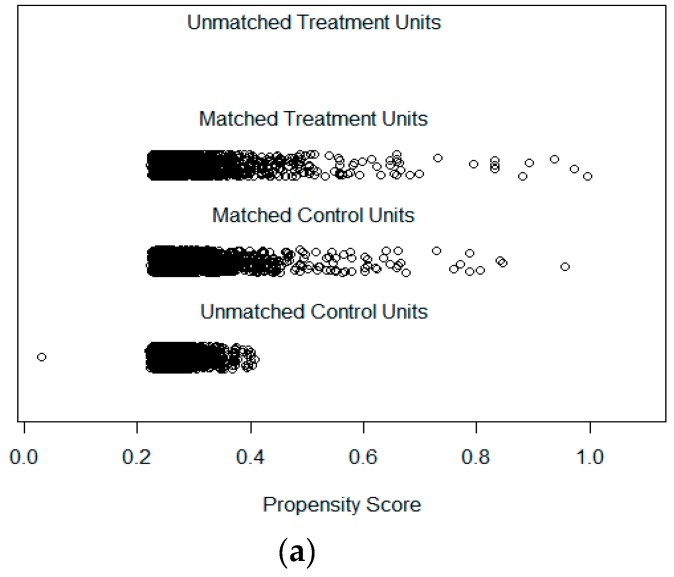

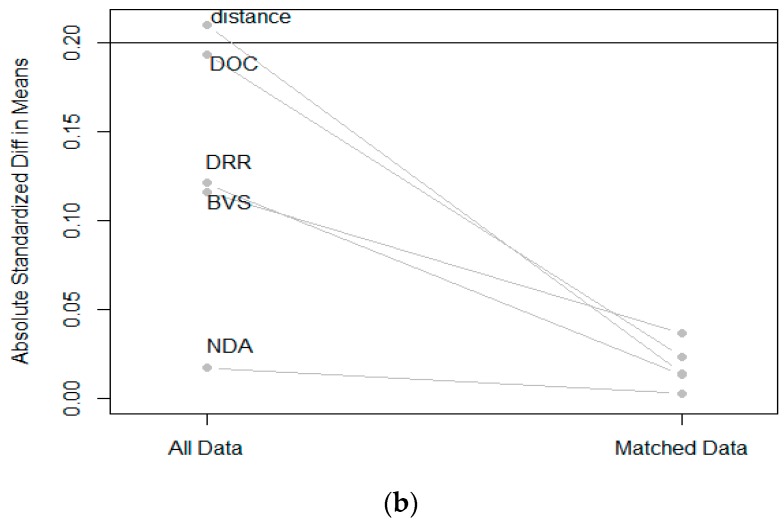
Distribution of Propensity Scores with the Experimental Data. (**a**) Diagnostics Graphical Plot, (**b**) Absolute Standard Difference Means.

Second, plots in Figure 3a show dots with a size proportional to their weight, which is also useful for weighting or subclassification. Meanwhile, the absolute standardized difference is helpful for comparing the mean of continuous variables between treatment groups, illustrated in Figure 3b. Those results were obtained in R with the package MatchIt [9]. The results of the absolute standardized difference suggested that the matched data achieved better covariate balance than all data before matching.

To diagnose the balance of the control-case data, we also compared the focus cases and matched control cases. Table 2 lists the statistics of the selected matched patient characteristics. The results provided empirical evidence that no statistically significant difference exists between those two groups of cases. To make the samples covariate balance, 2603 control cases were selected from the 7041 samples to compare with the package MatchIt [9].

### 3.2. Lorenz Curve of the Inequality Service

The OHC system associated the average influence of the reputation award on the doctors’ serviced patients and online views in each category, with the inequality measure (Gini coefficient) derived from the category’s Lorenz curve.

To diagnosis the difference of the cases in those two groups, we examined the data with Welch two sample *t*-test, as demonstrated in Table 3. Before matching, the means of patients are 1698 for the group control and 2680 for the focus cases. Since the null hypothesis is rejected, the alternative hypothesis is the true difference in means is not equal to 0. The results show that the mean of focus cases and that of the matched cases is significantly different.

**Figure 4:**
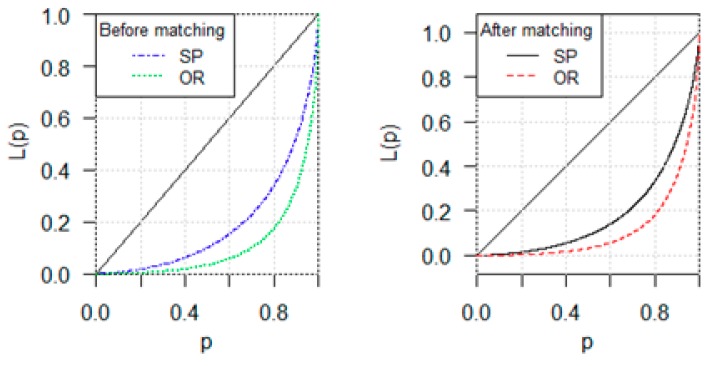
Lorenz Curve of the Empirical Experimental Data on Patient and Views Before Matching and After Matching. The Horizontal Axis Represents the Rank Percentile p of Severed Patients.

With the cases of control-case matching, the Gini coefficients of the empirical experimental data were compared among focus cases, control cases after matching and those before matching. We also compared the Gini of all the cases after matching and those of all the cases before matching, shown as in Table 4. Figure 4 deploys the Lorenz curve of the empirical experimental data on patients and views after matching and before matching.

The results in Table 4 show three essential facts. First, the number of views shows much higher inequality than that of patients for all the cases, the focus cases and the controls (no matter before matching or after matching). Second, the number of patients of focus cases shows higher inequality than those of controls, but the number of views of focus cases shows lower inequality than those of controls (both before matching and after matching). On patients, the difference of Gini coefficients between focus cases and controls after matching is 0.006 (=0.635–0.629), and that between focus cases and controls before matching is 0.031. On views, the difference of Gini coefficients between focus cases and controls after matching is −0.031 (=0.758–0.789), and that between focus cases and controls before matching is −0.022. Third, the number of patients of all the cases after matching show higher inequality than that of before matching, but the number of views of all the cases after matching show lower inequality than that of before matching. Moreover, the difference of inequality of health service between online views and offline serviced patients is 0.161 before matching in the 9644 cases, and 0.142 after matching for the 5206 cases.

### 3.3. Causal Effects of City-level on Services Inequality

We first identified the causal effects of cities of rich healthcare resources on online service and offline service with equation (13). Here we deduced the causal effect with the definition, which is different from the identification process of average treatment effect using regression. This is because the experimental data were provided with complete observations (not counterfactual) on the covariates. For Gini coefficients the specialty categories, 101 entities remained after filtering the NA values in the Gini coefficient table. The distribution of those Gini coefficients was deployed by the Gini coefficient of serviced patients and the views. For the Gini coefficient of serviced patients, 95% quantile of $SCGini_{j}$(SP) of focus cases is 0.721, which is 0.052 higher than that of the matched group. The 50% quantile of $SCGini_{j}$(SP) of focus cases is 0. 531, which is 0.025 higher than that of the matched group. And the average treatment effect of level-1 cities (the mean of $SCGini_{j}$(SP) of focus cases) is 0.470, which is 0.029 higher than that of the matched group. Similarly, for the Gini coefficient of online views, the 95% quantile of $SCGini_{j}$(OR) of focus cases is 0.840, which is 0.035 higher than that of the matched group. The 50% quantile of $SCGini_{j}$(OR) of focus cases is 0.642, which is 0.015 higher than that of the matched group. And the average treatment effect of level-1 cities (the mean of $SCGini_{j}$(OR) of focus cases) is 0.573, which is 0.016 higher than that of the matched group. Moreover, the difference between the average treatment effect of online views and that of offline serviced patients is 0.103 for the 101 specialties categories. In total, the results support the argument that the inequality of health service in level-1 cities is much higher (more serious) than that outside of those level-1 cities for different specialty categories. It also provides evidence that the patients are more likely to be aggregated in level-1 cities, and they are more likely to be served by the doctors.

## 4. Discussion

Although this paper is designed as a causal inference about the inequality of health service between online views and offline serviced patients for specialty categories, we also analyzed the associative relationship between those covariates and the (SCGini) responses of inequality of health service. With the cases before matching, we estimated the correlation between specialty category’s Gini coefficients and the other predictors (covariates), including the mean of the number of doctors’ articles across the specialty categories, mean in the degree of voted diversity, mean of doctors’ review rating and mean in doctors’ online contribution. The correlation between the Gini of the coefficient of serviced patients ($SCGini_{j}$(SP)) and the logarithm of $NDAMea_{j}$, $BVSMea_{j}$, $DRRMea_{j}$, and $DOCMea_{j}$ are relatively low (0.03, 0.05, 0.17 and 0.10, respectively). Similar results are depicted for the correlation between the logarithm of $SCGini_{j}$(OR) and the covariates. Based on these correlations, the variation of the response variable ($SCGini_{j}$(SP) and $SCGini_{j}$(OR)) may not be mainly explained by the covariates. As the results show, their R-Squared values are very low ($R^{2}$ = 2.5% and $R^{2}$ = 3.8%, respectively), illustrating that the model using ordinary regression is not interpretable to a substantial amount of variance in the dependent variable. The results support our argument that when the associative relationship with the constraints of strong related independent variables is not statistically significant, the causal inference method takes the advantages of non- significant related covariates by assigning treatment experiments on different units. The larger *NDAMea*, *DRRMea*, and *BVSMea* are, the inequality of $SCGini_{j}$(SP) would be lower, but a larger *DOCMea* would increase the inequality of $SCGini_{j}$(SP).

### 4.1. Principal Results

In the original data, the top four specialty categories of doctors’ serviced patients are gynecologic and pediatrics, five senses of Chinese traditional medicine (CTM), occupational disease and prosthodontics, with their average number of doctors’ serviced patients over 5000. However, CTM surgery, plant medicines, CTM infectious disease medicines, osteoporosis, and periodontitis are the lowest five specialty categories with a number of average doctors’ serviced patients under 800. The Gini coefficient of serviced patients ranges from 0.136–0.759 with a mean 0.564, which suggested that the inequality of health service in the online health community is relatively serious for the specialty categories. The total Gini coefficients of all the doctors in OHC are 0.632 for serviced patients and 0.774 for online views after control-case matching, and the Gini coefficient in level-1 cities is much higher (0.006 for serviced patients and −0.031 for online views) than those in the other cities.

Essentially, we should first realize that our empirical results cannot be used to explain all of the doctors’ specialties to serve patients but to interpret the causal effect of the city-level on the inequality of health service. As shown in Table 3 and Table 4, the causal effect of the city location on Gini coefficient was driven with the matched cases, which are the focus cases in level-1 cities with the potential control cities in the covariates of with the covariates as number of articles, breadth of service diversity, doctor’s review rating, doctor’s online contribution. Our findings show that, in various specialty areas, the average treatment effect of level-1 cities are different for doctors’ specialty categories. For the Gini coefficient of serviced patients in over 100 specialty categories, the average treatment effect of level-1 cities is 0.470, which is 0.029 higher than that of the matched group. Similarly, for the Gini coefficient of online views, the average treatment effect of level-1 cities is 0.573, which is 0.016 higher than that of the matched group.

Finally, we make specific recommendations for OHC managers to reduce the inequality in the distribution of doctors’ service delivery among specialty categories based on our findings. For example, platform managers should make an effort to reduce the service inequality, improving the referral system and assigning the patients to the matched doctors with the appropriate service diversity. Holding average influence constant, the association between the influence of the specificity diversity and the distributions service delivery was enhanced when the influence was spread more evenly across the doctors in the clinical title, rather than concentrated on a few doctors within the clinical title. For example, when the doctor encountered a not well-experienced disease case (with low votes for a few voted specialties), she/he may directly refuse to provide the online medical consultation service and suggested the patient to referral to another doctor or go to the hospital.

### 4.2. Limitations

Although the difference of inequalities between the units of cases from the level-1 cities and the others in OHC were reflected, more investigations need to be designed on the causality and policy evaluation. In the future, heterogeneity of the results should be considered for distinct groups of doctors who devoted different combinations of online contributions and online attendance. According to the scholarly commonsense of the coauthors, the samples may be grouped by their mean online contributions and online attendance values. As the samples did not completely conform to the standard normal distributions but were nevertheless supported, the mean value was used to represent the entire data set.

First, the number of doctors’ articles was collected at a specific time for this study. To further investigate the contribution of doctors’ articles, more properties of doctors’ articles could be abstracted in the future from the website, including the number of doctors’ articles written by themselves, number of doctors’ articles copies from others, the average count of words in a doctor’ articles, the average times of reviewing for a doctor’ articles, etc. Second, the measure of serviced patients used to rank experimental units when estimating the empirical Lorenz curve, and the corresponding Gini coefficient was subject to random error. This error could also lead to an incorrect ranking of experimental units that inevitably results in a curve that exaggerates the degree of diversity (variation) among doctors. Furthermore, all the data were collected from one single OHC, the Good Doctor website. Since the size of each individual doctor’ specialty was calculated in the patient voting process from 26–27 August 2017, there exists a bias in the measurement time interval. Moreover, propensity score matching (PSM) [32] in this study only accounted for observed (and observable) covariates, but the unobserved factors may influence assignment to treatment and outcomes while they cannot be accounted for in the matching procedure [33]. As PSM only controls for observed variables, there can still be hidden biases caused by latent variables after matching [34]. In the worst case, hidden bias may increase because matching on observed variables can unleash bias due to dormant unobserved confounders [35].

## 5. Conclusions

The causal inference method takes the advantages of non-significant related covariates, which assigns treatment experiments on different units. The research design in this paper avoids selection bias in the estimation of treatment effects. The Lorenz curve has been documented for a number of service diversities enrolled in OHCs. The distribution of the online service delivery (of patient virtual visits) across the physicians in a specialty category was characterized by a Lorenz curve in which the cumulative proportion of the volume of service delivery was plotted against the cumulative proportion of physicians in the same specialty category in the OHC. We designed a causal inference study with data on distributions of serviced patients and online views in over 100 distinct specialty categories on one of the largest OHCs in China. For the Gini coefficient of serviced patients in over 100 specialty categories, the average treatment effect of level-1 cities is 0.470, which is 0.029 higher than that of the matched group. Similarly, for the Gini coefficient of online views, the average treatment effect of level-1 cities is 0.573, which is 0.016 higher than that of the matched group. The results support the argument that the total Gini coefficient of all the doctors in the OHC shows that the inequality of health service is still very serious. The inequality of health service in level-1 cities is much higher (more serious) than that outside of those level-1 cities for different specialty categories. Such ac differential effect at the city-level on the Gini coefficient of health service delivery also provides evidence that the patients are more likely to be aggregated in level-1 cities, and they are more likely to be served by the doctors.

## Figures and Tables

**Table 1:** Variable Definitions and Measurements.

Variables	Definitions	Measurements
Dependent Variables		
$SCGini_{j}$(*SP*)	Specialty category’s Gini coefficient of serviced patients	Gini coefficient of doctors’ service delivery (serviced patients) for the doctors clustered in specialty category *j*
$SCGini_{j}$(*OR*)	Specialty category’s Gini coefficient of online views	Gini coefficient of doctors’ online views for the doctors clustered in specialty category *j*
Covariates		
$NDAMea_{j}$	Average number of articles	Average number of articles of the doctors clustered in specialty category *j*, and $NDA_{i}$ is the number of articles of the doctor *i*
$BVSMea_{j}$	Average breadth of service diversity	Average breadth of the voted specialties (from patient votes) of all the doctors clustered in specialty category *j*, and $BVS_{i}$ is the breadth of the voted specialties (from patient votes) of the doctor *i*
$DRRMea_{j}$	Average doctor review rating	Mean of the overall ratings in user reviews of the doctors clustered in the specialty category j (scoring from 1–5, already excluding 0), and $DRR_{i}$ is the number of the overall ratings in user reviews of the doctor *i*
$DOCMea_{j}$	Average doctor online contribution	Mean of doctors’ online contribution across the category’s doctors clustered in specialty category j, and $DOC_{i}$ is the number of doctors’ online contribution of the doctor *i*
Treatment variables: Divide the Samples Separately		
$CITY_{i}$	City level	A dummy variable $T_{i}\;$ with two levels: level-1 indicates doctors from resource-rich cities (Beijing and Shanghai); level-0 indicates doctors from other cities

**Table 2:** Statistics of the Selected Matched Patient Characteristics.

Variables	Focus Cases (*n* = 2603)	Matched Controls (*n* = 2603)	95% CI * in Difference After Matching	*p*-Value After Matching
NDA(i)	31.235	31.581	(−6.521; 7.215)	0.921
BVS(i)	9.244	9.376	(−0.067; 0.330)	0.194
DRR(i)	2.818	2.809	(−0.048; 0.029)	0.628
DOC(i)	34065.2	32516.6	(−4903.7; 1806.7)	0.366

*CI: confidence interval. NDA: Number of articles of each doctor; BVS: Breadth of the voted specialties; DRR: Number of the overall ratings in user reviews of each doctor; DOC: Number of doctors’ online contribution.

**Table 3:** Statistics of the Empirical Experimental Data (*n* = 2603).

	Mean of Focus Cases	Mean of Matched Controls	95% CI * in Difference	*p*-Value
Patients before matching	1698	2680	(−1158; −805)	<0.001
Patients after matching	2465	2680	(−436; 6)	0.056
Views before matching	1,065,312	2,191,087	(−1340802; −910749)	<0.001
Views after matching	1,771,188	2,191,087	(−695284; −144514)	0.003

*CI: confidence interval.

**Table 4:** Statistics (Gini Coefficients) of the Empirical Experimental Data.

	Gini of Focus Cases	Gini of Controls After Matching	Gini of Controls Before Matching	Gini of All the Cases after Matching	Gini of All the Cases Before Matching
SP	0.635	0.629	0.604	0.632	0.622
OR	0.758	0.789	0.780	0.774	0.783
Difference	0.123	0.16	0.176	0.142	0.161
*n*	2603	2603	7041	5206	9644

Note: SP—serviced patients; OR—online reviews.

## List of Abbreviations

ATE	Average treatment effect
OHC	online health community
SCGini	the specialty category’s Gini coefficient
O2O	online-to-offline
SP	served patients
OR	online reviews
NDA	the mean of the number of Doctors’ articles
BVS	the breadth of the voted specialties
DRR	the ratings in user reviews of the doctors
DOC	the contribution score for the doctors
CTM	Chinese traditional medicine
PSM	propensity score matching
